# Determination of Parabens and Their Metabolites in Seminal Plasma from Chinese Men by Ultra High Performance Liquid Chromatography Tandem Mass Spectrometry (UPLC-MS/MS)

**DOI:** 10.3390/toxics11020131

**Published:** 2023-01-29

**Authors:** Jing Dai, Linyuan Zhang, Jianfeng Xu, Fangda Peng, Zhijun Wu, Longlong Fu, Ying Guo, Bing Chang, Wenhong Lu, Chunguang Ding

**Affiliations:** 1 National Center for Occupational Safety and Health, National Health Commission of the People’s Republic of China, Beijing 102308, China; 2 National Institute for Occupational Health and Poison Control, Chinese Center for Disease Control and Prevention, Beijing 100050, China; 3 National Research Institute for Family Planning and WHO Collaborating Centre for Research in Human Reproduction, Beijing 100081, China; 4 National Health Commission Key Laboratory of Male Reproductive Health, Beijing 100730, China

**Keywords:** parabens, seminal plasma, metabolite, UPLC-MS/MS

## Abstract

Parabens are endocrine-disrupting chemicals (EDCs) that have estrogen-like activities and may cause male reproductive disorders. Here, we developed a method for the simultaneous determination of four parabens (MeP, EtP, n-PrP, n-BuP) and two metabolites (4-HB and 3,4-DHB) in human seminal plasma by UPLC-MS/MS. The method was used to analyze 144 seminal plasma samples from Chinese males. MeP, EtP, n-PrP, and 4-HB were the dominant compounds. MeP, EtP, and n-PrP were significantly correlated to each other. In addition, 4-HB was significantly correlated to MeP, EtP, n-PrP, and 3,4-DHB, respectively. The results provide direct evidence that parabens and their metabolites are widely distributed in the male reproductive system. The study presents the paraben metabolites levels in human seminal plasma for the first time.

## 1. Introduction

Parabens are homologous esters of p-hydroxybenzoic acid used for nearly 100 years due to their anti-microbial and anti-fungal properties [1]. Parabens were first reported in the 1920s to avoid microbial contamination and prevent degradation of the active ingredient [2]. After that, they have been used widely as preservatives in thousands of cosmetics, foods, and pharmaceutical products. Humans are usually exposed to parabens through skin absorption (cosmetic products), oral intake (food, medicines), and inhalation routes [3]. After absorption from the skin or intestine, parabens are very rapidly metabolized to 4-hydroxybenzoic acid (4-HB) by esterases in the liver. A significant proportion of these metabolites are excreted in the urine, including the free state metabolite (4-HB) and the conjugated state metabolites (glycine, glucuronic acid, and sulfuric acid conjugates of 4-HB [4]). Another part of the metabolite (4-HB) is further hydroxylated to 3,4-dihydroxybenzoic acid (i.e., protocatechuic acid) (3,4-DHB) [5,6], then excreted via urine. 

Parabens are endocrine-disrupting chemicals (EDCs) that have weak estrogenic activity [7,8]. Furthermore, several in vitro and in vivo studies examined the adverse effects of parabens on male reproductive fertility. In in vitro studies, Glander et al. [9] found that methylparaben decreased human sperm motility. Another study found that methyl-, ethyl-, propyl- and butylparaben showed spermicidal activity in in vitro assays [10]. Song et al. [11] reported that butylparaben exerts an inhibitory effect on the acrosomal enzyme acrosin and impairs sperm membrane function. Parabens have also been suggested to play a role in mitochondrial dysfunction and thus disturb sperm function [12]. In in vivo studies, the effects of paraben levels on reproductive outcomes remain controversial. Adverse effects on the reproductive system were observed in some animal experiments, evidenced by impaired function of the testes, decreased sperm counts, and low testosterone (T)levels [13,14,15]. On the contrary, under similar experimental conditions, some studies did not present any significant changes in reproductive organ weights, sperm motility, sperm count, and daily sperm production (DSP) [16,17]. Furthermore, studies found urinary levels of parabens were significantly associated with sperm DNA damage, abnormal sperm morphology, lower motility and testosterone levels, as well as XY18 and chromosome 13 disomy [18,19,20]. In addition to parent parabens, hydroxylated paraben metabolites also have shown significant association with some semen quality parameters [21]. 

The above research indicated that parabens have the risk of adverse effects on the male reproductive system, which could be reflected by the paraben levels in biological samples. Though the detection of parabens in biological samples has been reported in lots of literature [22,23,24,25], most of them focused on human blood and urine samples. Different from blood and urine, seminal plasma is composed of secretory products of the testis, epididymis, prostate, and seminal vesicle [26], which are the main organs that influence or induce the development of the entire male reproductive system. In comparison to blood and urine, seminal plasma can directly reflect the exposure of parabens in the male reproductive system. Furthermore, seminal plasma is a rare biological matrix in previous studies. Determination of paraben levels in seminal plasma allows us to find the concentration associations among seminal plasma, blood, and urine, which is significant to understand the tissue distribution and metabolism of paraben. Therefore, monitoring of parabens and their metabolites in seminal plasma has more biological significance. However, few studies focused on the paraben levels in seminal plasma. 

In this study, we developed and validated the UPLC-MS/MS method that allows the detection of four parabens (MeP, EtP, n-PrP, and n-BuP) and two metabolites (4-HB and 3,4-DHB) in seminal plasma simultaneously. The method has the advantages of simple pretreatment, high sensitivity, and good accuracy. Based on this method, we measured the contents of parabens and their metabolites in the seminal plasma samples of 144 Chinese men as well as analyzed the correlations between parent parabens and their metabolites. The study provides technical support for the safety evaluation of parabens and their metabolites in the male reproductive system in the future. 

## 2. Materials and Methods

### 2.1. Reagent and Chemical

Analytical standards of MeP, EtP, n-PrP, n-BuP, 4-HB, and 3,4-DHB were purchased from Sigma-Aldrich (St. Louis, MO, USA). Chlorzoxazone was purchased from National Institutes for Food and Drug Control (NIFDC, Beijing, China). β-glucuronidase (100,000 U/mL) was supplied by Sigma-Aldrich (St. Louis, MO, USA). Acetonitrile, methanol, ethyl acetate, ammonium acetate, and formic acid were supplied by Thermo Fisher Scientific (Houston, TX, USA). All the chemicals were of LC-MS or HPLC grade. Ultra-pure water was obtained from the Milli-Q system (Millipore, Bedford, MA, USA). 

### 2.2. Sample Collection and Preparation

A total of 144 seminal plasma samples were collected with help of the Department of Male Clinical Research, the Key Laboratory of Male Reproductive Health of National Health Commission of PRC, Human sperm bank, Research Institute of National Health Commission, Beijing, China. The inclusion criteria were the following: (1) being a healthy Chinese man aged 20–45 years with no prostatic disease, genito-urinary diseases, infectious diseases, or genetic disorders, (2) not receiving hormone treatment and unused illicit drugs or psychotropic drugs, (3) having myopia less than −6.00 D and no color blindness or color weakness, (4) the quality of semen does not meet the criteria for entering sperm banks. Sampling times are assumed to be random. Semen preparation of fresh ejaculate samples of semen were obtained via masturbation and then aliquots were taken for routine clinical evaluation of quality parameters of semen. Each semen sample was allowed to preserve at 37 °C until liquefaction fully occurred. The liquefied sample was immediately spanned at 4 °C for ten minutes at 2000× *g* and the supernatant was aliquoted into a 2 mL polyethylene tube. All samples were kept at −80 °C until use. Before sample collection, all participants signed informed consent. The present study was granted approval by the Medical Ethical Review Committee, National Institute for occupational health and poison control, Chinese center for disease control and prevention (201806).

### 2.3. Sample Preparation

After thawing, the 0.2 mL sample was aliquoted into a 1.5 mL microfuge tube, and 10 μL of the 200 ng/mL chlorzoxazone solution as an internal standard was precisely introduced. After this step, 20 μL of β-D-glucuronide enzyme solution was added to all samples. The tubes were vortexed for one minute and then incubated for 12 h at 37 °C. 

For the parent parabens analysis, the samples were extracted three times with 0.8 mL each of ethyl acetate (0.8 mL × 3). For each successive extraction, the mixture was shaken for 10 min and then centrifuged at 12000× *g* for 10 min. Supernatants were evaporated to dryness by a nitrogen stream. Finally, the concentrated samples were resuspended in 100 µL methanol. After filtration, 5 µL was injected into the LC-MS/MS system. 

For the paraben metabolites analysis, proteins were precipitated by adding 0.6 mL acetonitrile. As with the previous steps, the mixture was shaken for 10 min and then centrifuged at 12000× *g* for 10 min, where after 2 µL supernatant was injected into the LC-MS/MS system.

### 2.4. Apparatus and Operation Conditions

Samples were analyzed on a TQ-S micro (Waters, Millford, MA, USA) mass spectrometer (MS) coupled with an Acquity UPLC system (LC) (Waters, Millford, MA, USA). The analysis of 5 μL of the sample was injected into an analytical column (Waters Acquity UPLC BEH C_18_ column, 1.7 μm, 100 × 2.1 mm column; Waters, Millford, MA, USA). In addition, 0.1% formic acid in water constituted the mobile phase A and 0.1% formic acid in acetonitrile constituted the mobile phase B. The rate of flow was 0.4 mL/min. The gradient elution was applied and programmed as follows: the gradient started with 95% eluent A in 1.0 min and decreased linearly down to 60% in 0.5 min. This composition was held further for 4 min. Then, it decreased linearly again down to 10% in 0.5 min and held for 1 min before returning to 95% of eluent A immediately, followed by a re-equilibration for 2.5 min. The total run time for each sample analysis was 10.0 min.

The LC-MS system was conducted in negative ionization mode on a tandem quadrupole mass spectrometer (Acquity TQD; Waters, Milford, MA, USA) equipped with an ESI source. The optimal mass spectrometry settings were source temperature and capillary voltage, 150 °C and 2.5 kV, respectively. The rate of flow of cone gas was 50 L/h, and the rate of flow of desolvation gas was 850 L/h. Data analyses were performed with QuanLynx 4.0 software (Waters Corporation, Milford, MA, USA). The mass transitions of all the analytes are listed in Table 1.

### 2.5. Quality Control Procedure

A procedural blank and a spiked sample were run periodically for each batch of 20 samples to evaluate the possible contamination of samples and the accuracy of analytical procedures. The procedural blanks for target analytes were below the limit of detection (LOD). A series of calibration samples were assessed before and after the quality control samples and the unknown samples. Besides, solvent control was assessed once or twice in each batch. A typical sample batch per day consisted of 48 samples including the calibration samples.

### 2.6. Statistical Analysis

The statistical analysis (median, geometric mean (GM), average, minimum, and maximum value, 25th/75th percentile value) was performed using Excel 2019. Statistical analysis was conducted using the statistical package IBM SPSS 22.0. Correlations between analytes were assessed by Pearson correlation analysis. The significance level was set at 0.05. For the target analyte with a concentration below LOQ, LOQ/$\sqrt{2}$ was used for data analysis if its detection rate is more than 50%. All the figures were generated with Origin 2021 (OriginLab, Northampton, MA, USA).

## 3. Results

### 3.1. Mass and Chromatographic Condition Optimization

The optimized MS conditions were performed using direct infusion of a methanolic solution of negative ionization modes into the ESI source of the mass spectrometer, and parameters such as capillary and cone voltages were adjusted. The analytes were measured in the multiple reaction monitoring modes with specific transitions. The most intensive transition was used for quantification. 

**Figure 1:**
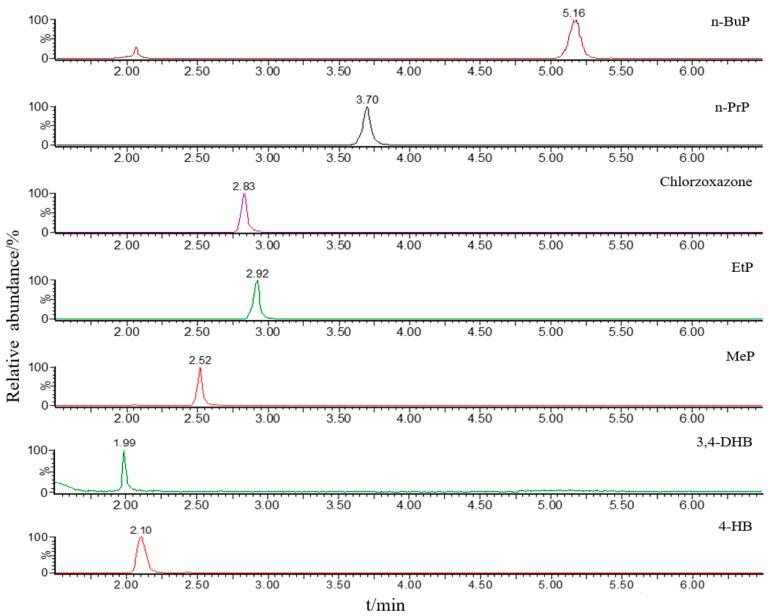
Chromatograms of four parabens (MeP, EtP, n-PrP, and n-BuP), two metabolites (4-HB and 3,4-DHB), and internal standard (Chlorzoxazone) at the concentration of 50 ng/mL obtained by MRM mode.

Given that the organic solvent and pH value of the mobile phase would affect ionization efficiency, two organic solvents (methanol and acetonitrile) and two additives (formic acid and acetic acid) were used for the optimization of the mobile phase. The ideal mobile phase was identified after several trials. Mobile phase A was H2O and mobile phase B was MeCN, both containing 0.1% formic acid. Besides, chromatographic parameters, such as the gradient of mobile phase, flow rate, column temperature, and injection volume, were all optimized for sample analysis. The chromatograms of parabens and their metabolites under optimized chromatographic and MS conditions are presented in Figure 1.

### 3.2. Method Validation

#### 3.2.1. Linearity, LODs, and LOQs

Calibration curves for each of the analytes in the method showed excellent linearity (weighed 1/X) with correlation coefficients greater than 0.994 (Table 2) over the studied concentration ranges. The limits of detection (LOD) and quantification (LOQ) of the method were calculated as the concentration giving a signal-to-noise ratio of three (S/N = 3) and ten (S/N = 10), respectively [27]. The precision and accuracy values of the LOQ for each analyte were within ±15%. LODs and LOQs of the method were presented in Table 2. LOQ is the lowest point of the measurement range.

#### 3.2.2. Precision and Accuracy

The intra- and inter-day precisions and accuracies for the parabens and their metabolites in seminal plasma are summarized in Table 3. Observed accuracy values were 91.49~110.09%; The precision of intra- and inter-assay ranged from 2.21 to 10.9% and from 0.45 to 11.7%, respectively.

### 3.3. Paraben Levels in Samples

**Figure 2:**
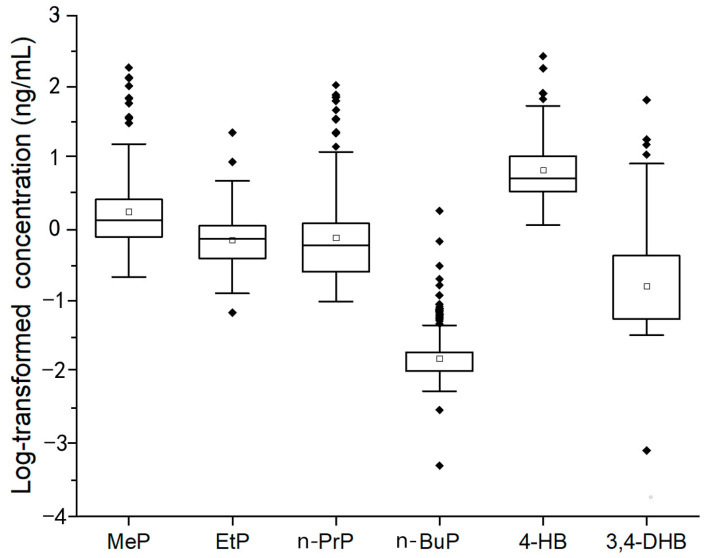
The levels of 4 parabens (MeP, EtP, n-PrP, and n-BuP) and their metabolites (4-HB and 3,4-DHB) in 144 seminal plasma samples (ng/mL).

The seminal plasma samples of 144 healthy Chinese men were determined by UPLC-MS/MS. The contents (expressed as geometric mean, percentile, mean, and range) and the detection rates (DRs) of target chemicals are summarized in Table 4 and Figure 2. 

For parent parabens, except n-BuP (with DR 28.47%), the detection rates of the other three parabens (MeP, EtP, and n-PrP) were 100%, indicating MeP, EtP, and n-PrP were more wildly existed in seminal plasma than that of n-BuP. The concentration ranges of MeP (0.22~185.72 ng/mL) and n-PrP (0.10 ~ 105.81 ng/mL) were much wider than that of EtP (0.07 ~ 22.85 ng/mL) and n-BuP (<LOD ~ 1.85 ng/mL). The GM concentrations of MeP, EtP, n-PrP, and n-BuP were 1.80 ng/mL, 0.74 ng/mL, 0.80 ng/mL, and 0.02 ng/mL, respectively.

For paraben metabolites, the detection rate of 4-HB was 100% while 3,4-DHB was only detected in 38.89% of samples, indicating 4-HB was a dominant metabolite of parabens in seminal plasma. The concentrations of 4-HB and 3,4-DHB ranged from 1.17 ~268.89 ng/mL and <LOD ~65.60 ng/mL, respectively. The GM concentrations of 4-HB and 3,4-DHB were 6.36 ng/mL and 0.15 ng/mL, respectively.

### 3.4. Correlations among Target Analytes

**Figure 3:**
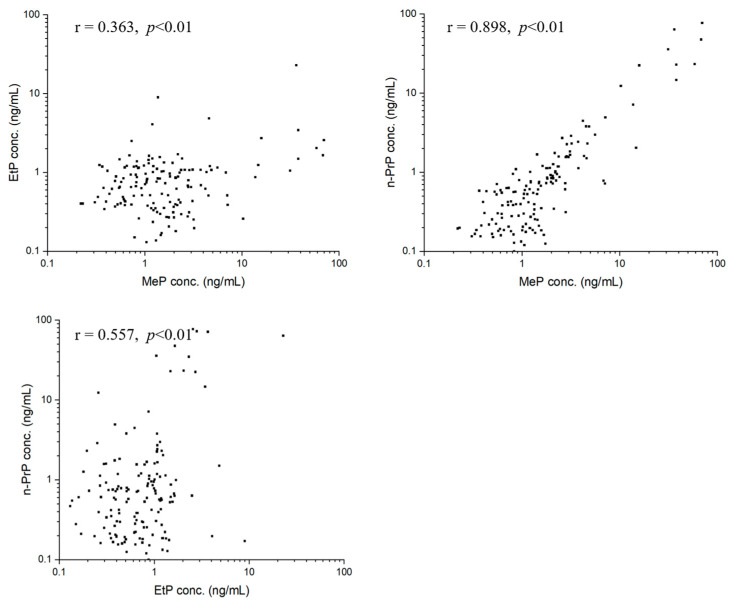
Correlations between MeP and EtP, MeP and PrP, EtP and n-PrP in seminal plasma.

**Figure 4:**
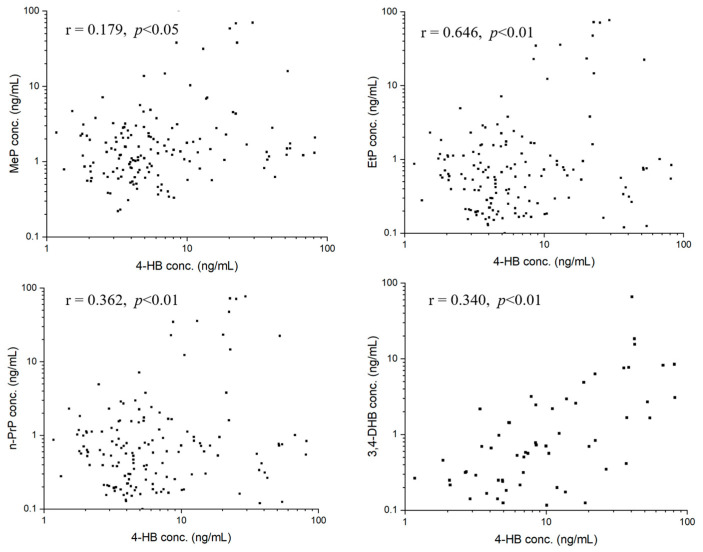
Correlations between 4-HB and MeP, 4-HB and EtP, 4-HB and n-PrP, 4-HB and 3,4-DHB in seminal plasma.

We performed statistical analyses to investigate correlations about target analytes using Pearson’s correlation analysis. For parent parabens, MeP, EtP, and n-PrP in seminal plasma were significantly correlated to each other (Figure 3). Specifically, a strong correlation between MeP and n-PrP concentrations was observed (r = 0.898, *p* < 0.01). Moderate correlations were observed between concentrations of MeP and EtP (r = 0.363, *p* < 0.01), and concentrations of EtP and n-PrP (r = 0.557, *p* < 0.01). For paraben metabolite of 4-HB, the concentration of each parent paraben had positive correlations with that of 4-HB, with the correlation coefficients of MeP (r = 0.179, *p* < 0.05), EtP (r = 0.646, *p* < 0.01), n-PrP (r = 0.362, *p* < 0.01) (Figure 4). The concentration of 3,4-DHB had a significant correlation (r = 0.340, *p* < 0.01) with that of 4-HB in 38.89% of detected samples (Figure 4).

## 4. Discussion

In this study, we developed a method to analyze four parabens and two metabolites of parabens simultaneously in human seminal plasma by UPLC-MS/MS. By this method, we detected the seminal plasma samples from 144 healthy Chinese men, with 100% detection rates for MeP, EtP, n-PrP, and 4-HB, 28.47% for n-BuP, and 38.89% for 3,4-DHB. 

In comparison to blood and urine, monitoring of parabens and their metabolites in seminal plasma has more biological significance, since it can directly reflect extent of male exposure to parabens in the male reproductive system, evaluate the potential impact of parabens on male reproductive system, as well as provide information helpful for metabolism research. In previous studies, parabens levels in human urine and plasma have been reported. In our study, we selected seminal plasma as the biological matrix, which is rare in other studies. Considering that differences in paraben levels among different biological matrices could reveal some information about tissue distribution and metabolism, we compared the median concentrations from human urine and plasma in the previous study and that of human seminal plasma samples in this study. First, the median concentrations of MeP, EtP, and n-PrP were lower in the seminal plasma in this study than that in urine in previous studies [28,29]. This is because parabens can be rapidly metabolized through phase I and phase II biotransformation, resulting in the rapid excretion of their parent forms and metabolized forms mainly through urine [29]. Second, compared with plasma [25,28], higher median concentrations of MeP, EtP, n-PrP, and n-BuP in the seminal plasma were observed in this study. This indicated that parabens could be accumulated more in reproductive systems than in plasma [30]. It is worth noting that n-PrP and n-BuP levels in seminal plasma were approximately 10-fold higher than in plasma. Furthermore, the harmful effects of n-PrP and n-BuP on the reproductive system in male mice have also been reported in the literature [13,14,15]. Therefore, the high accumulation of n-PrP and n-BuP in human seminal plasma raised our concern about the risk of harmful effects on the male reproductive system, which needs to be further evaluated. 

To estimate the internal exposure risk of parabens among different countries, we compared the median concentration and detection rate of each paraben in seminal plasma between Danish men reported previously [30] and Chinese men in this study. The detection rates and median concentrations both indicated MeP, EtP, and n-PrP more widely existed in seminal plasma samples in the Chinese population than that in Danish men. Specifically, compared with the detection rates of parent parabens (MeP, 55%; EtP, 63%; n-PrP, 95%) in the Danish population, higher detection rates (MeP, 100%; EtP, 100%; n-PrP, 100%) in seminal plasma of Chinese men in this study were observed. Furthermore, the median concentrations of MeP (1.40 ng/mL) and EtP (0.76 ng/mL) were both higher (1.41-fold for MeP and 5.43-fold for EtP) in seminal plasma in this study than that of Danish men (0.99 ng/mL of MeP and 0.14 ng/mL of EtP). For n-PrP, the median concentration of n-PrP (0.61 ng/mL) was close to the Danish men (0.68 ng/mL). For n-BuP, compared with the Danish population (median: 0.06 ng/mL, DR: 51%), both n-BuP levels and detection rate were much lower in the Chinese men (median: <0.015 ng/mL, DR: 28.47%). This could be because of the following reason: n-BuP is not included in National Standard Hygienic standards for uses of food additives (GB 2760) in China, resulting in fewer applications of n-BuP in food products from China. 

The study also performed a correlation analysis among parabens and their metabolites. First, significant positive correlations between MeP and n-PrP (r = 0.898, *p* < 0.01), MeP and EtP (r = 0.363, *p* < 0.01), EtP and n-PrP (r = 0.557, *p* < 0.01) were observed. The results reflected that these parabens (MeP, EtP, and n-PrP) probably have similar applications or are added to certain commodities at the same time. For example, MeP and n-PrP, the two parabens were used in combination in many commercial products (foods, pharmaceuticals, and cosmetics) [1,4,31]. Second, we also found significant positive correlations between the concentrations of metabolized paraben (4-HB) and three parent parabens, with correlation coefficients of 0.179 for MeP (*p* < 0.05), 0.646 for EtP (*p* < 0.01), and 0.362 for n-PrP (*p* < 0.01) (Figure 4), respectively, indicating that 4-HB in seminal plasma probably partly came from the metabolism of MeP, EtP and n-PrP. Third, for paraben metabolites, the concentrations of 4-HB and 3,4-DHB are significantly correlated (r = 0.340, *p* < 0.01). This is because hydroxylation of 4-HB causes the formation of 3,4-DHB. 

Previous researchers generally focused on the parent parabens while little attention was paid to their metabolites. This is the first study to report concentrations of paraben metabolites (4-HB and 3,4-DHB) in seminal plasma. The detection of metabolites suggests that the harmful effects of these metabolites need to be further evaluated. However, the safety of 4-HB is controversial. Although studies have shown that 4-HB has low antiandrogenic efficacy (if any) and lacks a statistically significant inhibitory effect [32], its estrogen activity may also increase the risk of incomplete masculinization, resulting in decreased sperm quality [7,33,34,35]. The two paraben metabolites have been reported as potential endocrine disruptors by USEPA (U.S. Environmental Protection Agency), suspected to affect male reproduction, and need more attention.

## 5. Conclusions

In this study, a simple, sensitive method was established for the simultaneous determination of four parent parabens and two metabolized parabens in human seminal plasma samples by ultra-high performance liquid chromatography tandem mass spectrometry (UPLC-MS/MS). The method was applied to detect 144 seminal plasma samples from Chinese males. Our results showed high detection rates (100%) for MeP, EtP, and n-PrP, which suggested that the adults in China were exposed to parabens widespread. MeP, EtP, and n-PrP in seminal plasma were significantly correlated to each other. Moreover, we found 4-HB and 3,4-DHB in seminal plasma samples. To the best of our knowledge, this is the first study to report paraben metabolites in seminal plasma. We also found significant positive correlations among the 4-HB and MeP, EtP, n-PrP, and 3,4-DHB. The results showed the non-negligible internal exposure levels of parent and metabolized parabens existing in seminal plasma in Chinese adults, which raised concern about the risk of harmful effects on the human reproductive system. However, both the adverse effects of parent and metabolized parabens lack in vivo data. Our results indicated that further analyses are required to examine the long-term effects of parabens and their metabolites on the male reproductive system.

## Figures and Tables

**Table 1:** MS conditions and retention times for each detected compound.

No.	Compound	Parent Ion	Product Ion	Cone Voltage/V	Collision Energy/eV	Ionization Mode	Retention Time/Min
		(m/z)	(m/z)				
1	MeP	150.76	91.95	6	18	[M-H]-	2.52
2	EtP	165.03	92.08	20	22	[M-H]-	2.92
3	n-PrP	179.11	92.05	22	24	[M-H]-	3.70
4	n-BuP	193.06	91.99	2	22	[M-H]-	2.07
5	4-HB	137.06	93.57	32	15	[M-H]-	2.08
6	3,4-DHB	151.80	80.96	14	18	[M-H]-	1.99
7	Chlorzoxazone	168.02	131.98	44	18	[M-H]-	2.83

**Table 2:** Linear equations, measurement ranges, correlation coefficients, limits of detection of parabens and their metabolite.

No.	Compound	Linear Equation	Measurement Range/(ng/mL)	r^2^	LOD/(ng/mL)
1	MeP	y = 0.2281x − 0.2983	0.06–100	0.9978	0.019
2	EtP	y = 0.3044x + 0.0494	0.07–100	0.9993	0.021
3	n-PrP	y = 0.3968x + 0.2257	0.05–100	0.9985	0.016
4	n-BuP	y = 0.4954x + 0.0185	0.045–5	0.9992	0.015
5	4-HB	y = 0.8092x − 0.7795	0.07–100	0.9991	0.023
6	3,4-DHB	y = 0.1043x + 0.1828	0.25–100	0.9947	0.081

**Table 3:** Spiked recoveries and RSDs of the detected compounds at 3 levels in seminal plasma.

No.	Compound	Added (ng/Ml)	Recovery/%	RSDs/%	
				Intra-Assay (*n* = 6)	Inter-Assay (*n* = 3)
1	MeP	0.06	95.92	7.42	2.98
		20	104.55	4.71	3.81
		80	91.49	2.21	0.55
2	EtP	0.07	90.30	8.08	8.45
		20	101.33	3.71	4.08
		80	88.78	7.53	4.83
3	PrP	0.05	95.05	5.82	6.61
		20	110.09	3.14	5.74
		80	95.02	4.46	5.8
4	BuP	0.045	93.76	9.46	2.27
		1	109.77	2.73	4.79
		4	97.79	3.93	4.92
5	4-HB	0.07	91.89	10.27	6.36
		20	109.47	3.98	11.2
		80	108.09	2.32	11.7
6	3,4-DHB	0.25	98.7	6.09	8.68
		20	106.93	2.32	10.6
		80	107.55	4.23	10.3

**Table 4:** Concentrations (ng/mL) of parabens and their metabolites in seminal plasma samples (*n*= 144).

Analyte	GM ^a^	Percentiles			Mean	Range	DR ^b^ (%)
		25th	Median	75th			
paraben							
MeP	1.80	0.78	1.40	2.63	8.03	0.22~185.72	100.00
EtP	0.74	0.41	0.76	1.15	1.18	0.07~22.85	100.00
n-PrP	0.80	0.26	0.61	1.32	4.98	0.10~105.81	100.00
n-BuP	0.02	<LOD	<LOD	0.02	0.04	<LOD~1.85	28.47
paraben metabolite							
4-HB	6.36	3.37	4.92	9.69	13.16	1.17~268.89	100.00
3,4-DHB	0.15	<LOD	<LOD	0.32	1.26	<LOD~65.60	38.89

a—GM = geometric mean; b—DR = detection rate.

## Data Availability

The data presented in this study are available on request from the corresponding author.
